# A trial comparing continuous positive airway pressure (CPAP) devices in preterm infants

**DOI:** 10.1038/s41372-020-0690-5

**Published:** 2020-05-20

**Authors:** Carl H. Backes, Jennifer N. Cooper, Jennifer L. Notestine, Crystal M. Alfred, Molly K. Ball, Brian K. Rivera, Jane M. Lamp, Laura Marzec, Michael R. Stenger, Mohannad Moallem, Randy R. Miller, Apurwa Naik, Lindsey J. Beer, Christopher R. Howard, Stephen E. Welty, C. Peter Richardson, Noah H. Hillman, John A. F. Zupancic, Larissa I. Stanberry, Thomas N. Hansen, Charles V. Smith

**Affiliations:** 1 Center for Perinatal Research, The Abigail Wexner Research Institute at Nationwide Children’s Hospital, Columbus, OH USA; 2 Department of Pediatrics, The Ohio State University Wexner Medical Center, Columbus, OH USA; 3 The Heart Center, Nationwide Children’s Hospital, Columbus, OH USA; 4 Center for Surgical Outcomes, The Abigail Wexner Research Institute at Nationwide Children’s Hospital, Columbus, OH USA; 5 Division of Epidemiology, The Ohio State University College of Public Health, Columbus, OH USA; 6 Department of Pediatrics, Mt. Carmel St. Ann’s Hospital, Westerville, OH USA; 7 OhioHealth Grant Medical Center, Columbus, OH USA; 8 Central Ohio Newborn Medicine, Columbus, OH USA; 9 Seattle Children’s Research Institute, Seattle Children’s Hospital, Seattle, WA USA; 10 Seattle Children’s Neonatology Program, CHI Franciscan Health, Tacoma, WA USA; 11 SSM Health, Cardinal Glennon Children’s Hospital, St. Louis University, St. Louis, MO USA; 12 Division of Newborn Medicine, Harvard Medical School, Boston, MA USA; 13 Department of Neonatology, Beth Israel Deaconess Medical Center, Boston, MA USA; 14 Minneapolis Heart Institute Foundation, Minneapolis, MN USA

**Keywords:** Translational research, Paediatrics

## Abstract

**Objective:**

To test the hypothesis that infants born <30 weeks’ gestation supported by Seattle-PAP will have lower rates of continuous positive airway pressure (CPAP) failure than infants supported with conventional, Fisher&Paykel-CPAP (FP-CPAP).

**Study design:**

Randomized trial (3/2017-01/2019) at 5 NICUs. The primary outcome was CPAP failure; subgroup analyses (gestational age, receipt antenatal corticosteroids) were performed.

**Results:**

A total of 232 infants were randomized. Infants in the Seattle-PAP and FP-CPAP groups had mean gestational ages of 27.0 and 27.2 weeks, respectively. We observed no differences in rates of treatment failure between Seattle-PAP (40/112, 35.7%) and FP-CPAP (38/120, 31.7%; risk difference, 4.1%; 95% CI, −8.1–16.2; *P* = 0.51). Subgroup analysis indicated no differences in rates of CPAP failure. We observed no differences between the two groups in frequencies of adverse events or duration of respiratory support.

**Conclusions:**

Among infants born <30 weeks’ gestation, rates of CPAP failure did not differ between Seattle-PAP and FP-CPAP.

## Introduction

Respiratory distress in the perinatal period is common among preterm infants, particularly among those born at <30 weeks of gestation [1]. The American Academy of Pediatrics has endorsed the use of continuous positive airway pressure (CPAP) among preterm infants with respiratory distress [2], based on lower rates of the combined outcome of bronchopulmonary dysplasia (BPD) or death [3]. CPAP failure, defined as the need for tracheal intubation and mechanical ventilation, is common among preterm infants <30 weeks of gestation, with failure rates approaching 45–50% in large clinical trials [4–7]. In developing countries and resource-limited facilities in which intubation and mechanical ventilation is not available, CPAP failure is associated with greater mortality [8]. Thus, preventing CPAP failure remains a high priority among health care providers caring for preterm infants [9]. These observations led to the design and development of a novel, low-cost, CPAP system—Seattle-PAP.

**Fig. 1:**
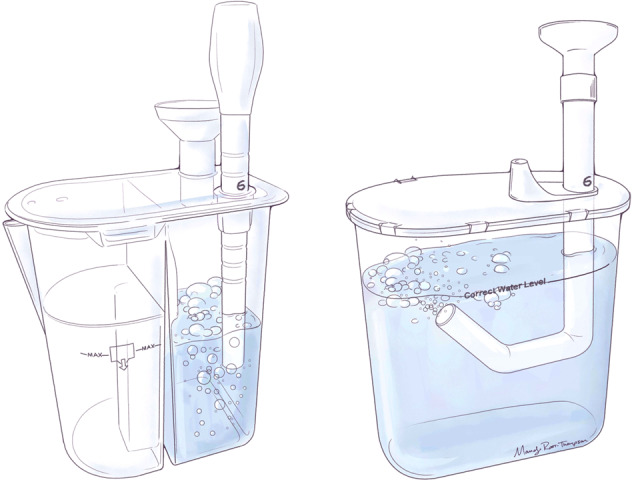
Illustration of Fisher & Paykel (left) and Seattle-PAP (right) bubble CPAP generators. The expiratory limb of the circuit is oriented vertically into the water seal of the bubbler apparatus (0°) in Fisher & Paykel, while the expiratory limb of the circuit in Seattle-PAP is maintained at 135°.

In traditional bubble CPAP systems, the expiratory limb of the circuit is oriented vertically into the water seal of the bubbler apparatus (0°). In contrast, the expiratory limb of the circuit in Seattle-PAP is maintained at 135° (Fig. 1), which leads to fluctuations in airway pressure that are notably different than with traditional bubble CPAP systems [8, 10–12]. In preclinical studies, Seattle-PAP resulted in higher arterial oxygen pressure (PaO_2_) levels and markedly lower (50%) work of breathing, than conventional CPAP systems [11]. In addition, in a small, single-center study of preterm infants (average gestational age of 29 weeks), breathing effort was lower with Seattle-PAP than with conventional Fisher & Paykel bubble CPAP (FP-CPAP), with no indications of increased rates of adverse events; however, that study was not designed to test differences with Seattle-PAP on clinical outcomes [12]. Despite the strength of the pre-clinical and early clinical studies, few centers have extensive experience with Seattle-PAP. We therefore conducted a multicenter, randomized controlled trial (RCT) to test the hypothesis that premature infants (<30 weeks of gestation) supported by Seattle-PAP would exhibit lower rates of CPAP failure than would neonates supported with FP-CPAP.

## Patients and methods

### Participating institutions, study design, and oversight

The Nationwide Children’s Hospital (NCH) IRB approved the trial (#16–00678); review was ceded to the NCH IRB by Riverside Methodist Hospital, Grant Medical Center, and The Ohio State University Wexner Medical Center. The IRB at Mt. Carmel St. Ann’s Hospital granted separate institutional approval (#170817–2). The facilities routinely care for premature infants with respiratory distress, using FP-CPAP as the primary noninvasive respiratory support modality. Participating sites did not use Seattle-PAP prior to study commencement. The trial was registered with ClinicalTrials.gov (NCT03085329). An external safety and efficacy monitoring board (DSMB) conducted regular reviews of patient safety (treatment A vs. treatment B) using compiled data summaries. Details regarding the trial study design and protocol have been published previously [13].

### Eligibility and recruitment

Infants were eligible for inclusion if delivered at gestational ages between 22^0/7^–29^6/7^ weeks and if they were candidates for noninvasive respiratory support, either as: 1) initial support between birth and 72 h of age; or 2) following the initial extubation and withdrawal of mechanical support during the first 72 h of life. Infants were excluded if their postnatal ages were >72 h at the time of extubation, they had a known congenital anomaly (including any airway abnormality [e.g., Pierre-Robin sequence and cleft lip and palate]) that might adversely affect breathing, had a known genetic anomaly (e.g., Trisomy 21), or if maximal intensive care was not being provided. Infants were recruited at a large US pediatric academic medical center with five participating neonatal intensive care units (NICUs) that share guidelines in the care and management of preterm infants. Infants transported from outside institutions, if meeting eligibility criteria, were also approached. Study team members obtained written, informed consent from parents or guardians of eligible infants; antepartum consent was sought, when possible. If antepartum consent was not obtained, parents/guardians of eligible infants were approached as soon as possible following delivery, up to 72 h’ postnatal age.

### Randomization and blinding

Upon receipt of consent and verification of eligibility, treatment assignments were performed with the use of a secure study website. Participants were assigned randomly to treatment with either Seattle-PAP or FP-CPAP. Due to the nature of the intervention, blinding as to the assigned treatment arms was not possible with respect to the treatment teams. Blinding was achieved for data analysis purposes by assigning generic identifiers among trial participants prior to data transfer. We did not stratify by site, as treatment of infants and additional aspects of care were based on shared guidelines among the five participating NICUs [13, 14].

### Treatment strategies

#### Delivery room management

Among mother–infant dyads consented antenatally, the assigned CPAP device (Seattle-PAP or FP-CPAP) was initially offered to infants as the primary respiratory modality; however, INSURE (INtubate, SURfactant, Extubate) was permitted [9]. Delivery room management (e.g., criteria for intubation), followed international guidelines, including those of the Neonatal Resuscitation Program [15, 16]. For mother–infant dyads that consented postnatally, randomization to the assigned CPAP device was performed at the time of the first decision to use noninvasive support.

#### Guidelines for extubation to CPAP from mechanical ventilation

Weaning from mechanical ventilation was based on established guidelines across participating NICUs [13, 14]. Extubation was recommended within 24 h of meeting all the following criteria: fraction of inspired oxygen (FiO_2_) of 0.30 or less with oxygen saturation as measured by pulse oximetry (SpO_2_) of 92% or greater, mean airway pressure of 8 cm H_2_O or less with hemodynamic stability, and receiving caffeine (either a loading dose of 20 mg per kilogram body weight or a maintenance dose of 5 mg per kilogram) [17]. While the guidelines emphasize administration of exogenous surfactant therapy for infants on mechanical ventilation with FiO_2_ > 0.40, use was at the discretion of the attending provider.

#### Guidelines for intubation

Infants who could not be maintained with their assigned CPAP device were intubated and ventilated; the originally assigned intervention was resumed after extubation. Consistent with previous studies, intubation was recommended if: 1) more than two episodes of apnea requiring bag-mask ventilation were encountered in a 24 h period or more than six episodes requiring any intervention within a 6 h period; 2) FiO_2_ of 0.50 or greater to maintain SpO_2_ of 88%; 3) cardiovascular instability; 4) as recommended by treating health care provider [17, 18]. Among infants with apnea, administration of caffeine was stressed in the guidelines, but at the discretion of the attending provider. All participating NICUs placed an emphasis on minimizing laboratory studies; thus, arterial or venous blood gases (pH, carbon dioxide) were not included in the recommendations for reintubation [14]. Adherence to treatment guidelines were monitored and reported [13].

#### Guidelines for weaning from CPAP

Based on expert opinion, for infants requiring FiO_2_ > 0.21, weaning from the assigned CPAP device to any nasal cannula, including high-flow nasal cannula (HFNC), was not endorsed prior to 32 weeks’ postmenstrual age [19]. Failure to maintain target oxygen saturations on room air without respiratory support prompted return of the infant to the assigned CPAP device.

#### Respiratory care for infants on CPAP

In the absence of clear data to guide the practice of evidence-based medicine, and reflecting current practice patterns, the use of chin-straps and pacifiers, and body positioning (prone, lateral), were not specified [9]. To avoid erosive damage and minimize the risk of subsequent nasal deformities, nasal prongs were chosen that: 1) fit securely in the infant’s nares; 2) avoided pinching the septum or blanching of the nares. CPAP was delivered though either short bi-nasal prongs or a nasal mask [20]. To decrease the risk of nasal injury, prongs, and masks were alternated every 3–6 h [21]. The frequency of gentle suctioning of the nasal cavities, oropharynx, and stomach was recommended every 3–4 h; however, guidelines stressed that suctioning needs should be dictated by clinical assessment (e.g., increased oxygen requirement, increased work of breathing). In most cases, a 6 French (Fr) catheter was used for suctioning, with a continuous suction pressure of −80 mmHg. Additional details on treatment guidelines for the present study have been previously published [13].

### Study outcomes

The primary outcome was CPAP treatment failure, defined by the following: 1) tracheal intubation within 72 h for surfactant administration after initiation of bubble CPAP and then not extubated by 72 h; 2) tracheal intubation or support with biphasic CPAP (synchronized nasal intermittent positive pressure ventilation, SiPAP) after 72 h post-delivery and up to 32 weeks gestational age; 3) inability to sustain SpO_2_ of at least 90%, despite noninvasive respiratory support of 8 cm H_2_O bubble CPAP, and FiO_2_ > 0.40 for more than 1 h. Consistent with previous studies [18], 72 h post-delivery was chosen to provide a window for surfactant administration. As adjudicated by an independent investigator, tracheal intubations for surfactant delivery (within 72 h post-delivery) and non-respiratory issues (e.g., surgery for retinopathy of prematurity) were not considered treatment failures.

Pre-specified secondary outcomes included reasons for treatment failure, time (hours) to treatment failure after randomization, days of mechanical ventilation (synchronized intermittent mandatory ventilation [SIMV] or high-frequency oscillatory ventilation [HFOV]) after trial entry, days of any positive pressure (SIMV, HFOV, SiPAP, CPAP, and HFNC), days of oxygen therapy after trial entry, days on room air after trial entry, days to achieve full enteral feeds (>130 mL/kg/day) after trial entry, days to achieve full-suck feeding (>130 ml/kg/day) after trial entry, and weight at discharge. Differences in CPAP failure rates among infants who had antenatal consent and were started on CPAP at birth were examined. The complete list of pre-specified secondary outcomes is provided in the published study protocol [13]. Serious adverse events were defined in the previously-published study protocol [13]. These events were reported to the data safety and monitoring board (DSMB) as they occurred. Data were collected until death or discharge home.

### Statistical analysis

Based on data from previous clinical trials of preterm infants using CPAP, we estimated a treatment failure rate of 50% in the control arm (FP-CPAP) [4–6]. From earlier studies, we estimated that treatment failure rates of Seattle-PAP to be 30% [8, 12]. As multiples were to be randomized as a set to the same study arm, the sample size estimate was inflated by 1.12, to allow for the effect of clustering. Accounting for two interim analyses utilizing a Haybittle–Peto stopping guideline set at *P* < 0.001, for the study to have 80% power with a two-tailed type I error of 0.05, a sample size of 230 infants was required.

All analyses were performed on an intention to treat basis. Continuous data are expressed as means with standard deviations or as medians with interquartile ranges (IQR), whereas categorical variables are expressed with frequencies and proportions. The primary outcome and other binary outcomes occurring in at least 5% of patients were analyzed using Pearson chi-squared tests. Less common binary outcomes were compared using Fisher’s exact tests. Risk differences were calculated, along with their 95% confidence intervals.

Normally distributed continuous outcomes were compared using the Student’s *t* test, whereas Wilcoxon rank-sum tests were used to compare continuous outcomes with skewed distributions. Planned analyses were performed to assess treatment effect heterogeneity across several clinical characteristics known to be associated with CPAP treatment failure: gestational age, birth weight, and exposure to antenatal corticosteroids [22]. Treatment effect heterogeneity was tested by evaluating the significance of interactions between the factors of interest and treatment arm in log binomial regression models that included the factor, treatment arm, and their interactions. Regression models were estimated using generalized estimating equations, to account for the inherent correlations expected with multiples.

In a sensitivity analysis, in order to account for the correlation in outcomes expected within multiples and the stratification of randomization by gestational age, we fit marginal regression models. Robust standard errors were estimated, and all models included gestational age (22^0/7^–<27 weeks’ gestation; 27–29^6/7^ weeks’ gestation). Log binomial regression models were fit for dichotomous outcomes, negative binomial regression models for count outcomes, linear regression models for continuous outcomes, Cox proportional hazards models for time to event outcomes, and cumulative logistic regression models for ordinal outcomes. In addition, we performed sensitivity analyses following the exclusion of either: 1) infants with a delayed (after randomization) diagnosis of major congenital anomaly; or 2) infants with protocol deviations, whether the deviation occurred before or after primary outcome determination. Analyses were performed using Statistical Analysis Software (SAS) Enterprise Guide version 7.15 (SAS Institute Inc., Cary, NC, USA).

### Data and safety monitoring committee

Prior to trial commencement, a DSMC was appointed and consisted of four health care providers with expertize in neonatology, resuscitation, and clinical trial statistics. *A priori* stopping rules for adverse events and efficacy were established. Interim analyses were conducted by the DSMB following completion of study of 25 and 110 infants, respectively. The analyses compared the two groups with respect to efficacy, safety, and futility. The interim analyses were completed in June 2017 and January 2018. Based on these analyses, the DSMB recommended that the trial continue without modification.

## Results

### Study population

**Fig. 2:**
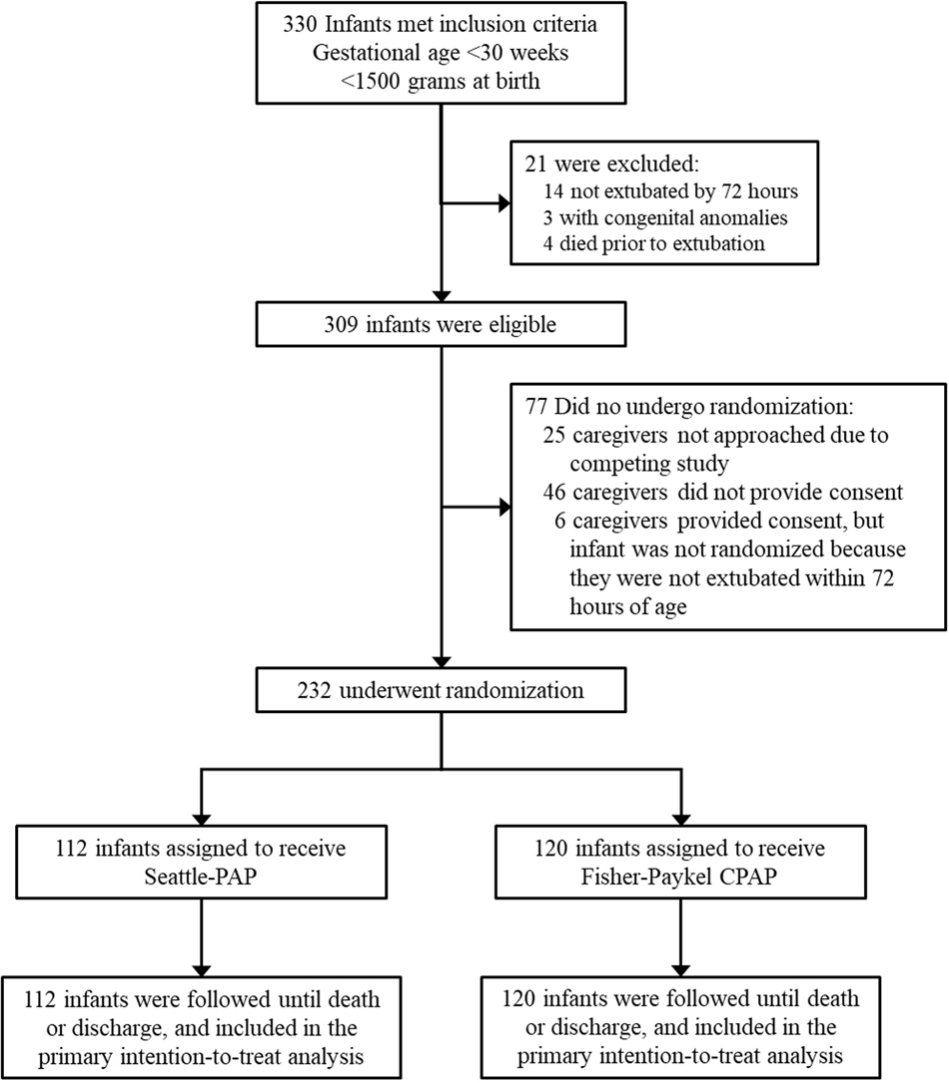
Screening, randomization, and study completion. From March 24th, 2017 to January 5th, 2019, a total of five NICUs screened 330 infants who met the inclusion criteria, of whom 309 were eligible and 21 were excluded. The caregivers of 25 infants were not approached for informed consent, the caregivers of 46 did not provide consent, and six caregivers provided consent, but the infant did not undergo randomization after failing to extubate within 72 h of life. A total of 232 infants were randomized and enrolled. CPAP denotes continuous positive airway pressure.

The trial took place from March 24th, 2017 to January 5th, 2019. A total of 232 premature infants underwent randomization (112 to the Seattle-PAP group and 120 to the FP-CPAP group, (Fig. 2). Infants in the Seattle-PAP and FP-CPAP groups had mean gestational ages (GA) of 27.0 and 27.2 weeks, respectively. Among eligible infants whose parents/guardians were approached for study participation and were extubated <72 h of age, 83.5% (232/278) were consented and randomized. Baseline maternal and infant characteristics (Table 1) were well balanced between the groups, although the proportions of male infants and mothers self-identified as black or African American were higher in the Seattle-PAP group than in the FP-CPAP group.

**Table 1:** Study population characteristics.

Maternal characteristics	Seattle-PAP (*n* = 103)	FP-CPAP (*n* = 105)
Primigravida—no. (%)	34 (33.0)	40 (38.1)
Age—years	28.8 ± 6.2	28.3 ± 6.0
Race or ethnicity^a^—no. (%)		
Caucasian	71 (68.9)	78 (74.3)
Black or African American	28 (27.2)	16 (15.2)
Hispanic	0 (0)	5 (4.8)
Asian or Pacific Islander	2 (1.9)	0 (0)
Multiracial	9 (8.7)	5 (4.8)
Other	2 (1.9)	2 (1.9)
Unknown	0 (0)	1 (1.0)
Maternal marital status—no. (%)^b^		
Single	50 (48.5)	53 (50.5)
Married	53 (51.5)	50 (47.6)
Mother in pre-term labor—no. (%)	62 (60.2)	63 (60.0)
Rupture of membranes—no. (%)	38 (36.9)	44 (41.9)
Period prior to delivery (hours)^c^	120 (0–1224)	71 (0–1056)
Diagnosis of chorioamnionitis^d^—no. (%)	25 (24.3)	25 (23.8)
Antenatal glucocorticoids received—no. (%)	93 (90.3)	101 (96.2)
Number of doses of antenatal corticosteroids	2 (1–4)	2 (1–4)
Delivery method—no. (% of infants)		
Vaginal	32 (28.6)	42 (35.0)
Cesarean section	80 (71.4)	78 (65.0)

Infant Characteristics	Seattle-PAP (*n* = 112)	FP-CPAP (*n* = 120)
Gestational age (weeks)	27.0 ± 1.8	27.2 ± 1.7
<27 wk—no. (%)	36 (32.1)	39 (32.5)
27–<30 wk—no. (%)	76 (67.9)	81 (67.5)
Birth weight (grams)	990 ± 253	1020 ± 254
Male sex (%)—no. (%)	70 (62.5)	59 (49.2)
Multiple gestation (%)—no. (%)	25 (22.3)	34 (28.3)
Intubated in the delivery room—no. (%)	49 (43.8)	44 (36.7)
Surfactant treatment—no. (%)		
In the delivery room	31 (27.7)	38 (31.7)
At any time	74 (66.1)	73 (60.8)
Apgar scores at five minutes, median (IQR)^e^	7 (5–8)	7 (6–8)
Postnatal age at randomization, median (IQR)—hours	8 (0–17.8)	10 (0–20.0)
Fraction of inspired oxygen at randomization, median (IQR)	25.0 (21–30)	24.5 (21–30)
Age at randomization, median (range)—hours	8 (0–60)	10 (0–70)
Caffeine received in first 24 h of life—no. (%)	111 (99.1)	119 (99.2)

No significant differences between the treatment groups for any characteristics were observed.

Values reported are means ± SD or Median (Interquartile Range *IQR*). All other variables are reported as frequencies and percentages.

^a^Race and ethnicity were self-reported.

^b^Not indicated in two cases from FP-CPAP group.

^c^Duration of rupture of membranes was not reported in 12 cases.

^d^Clinical diagnosis of chorioamnionitis.

^e^APGAR scores at 5 min not reported (*n* = 2).

### Primary outcome

Rates of CPAP treatment failure did not differ between Seattle-PAP (40/112, 35.7%) and FP-CPAP (38/120, 31.7%; risk difference, 4.1 percentage points; 95% CI, −8.1–16.2; *P* = 0.51). Treatment effect heterogeneity by gestational age, birth weight, or exposure to antenatal corticosteroids was not observed (Table 2).

**Table 2:** Characteristics of patients who experienced treatment failure.

Characteristics	Seattle-PAP (*n* = 112)	FP-CPAP (*n* = 120)	*P* Value
Gestational age			0.06
<27 weeks (*n* = 36, 39)	26 (72.2)	31 (79.5)	
27–<30 weeks (*n* = 76, 81)	14 (18.4)	7 (8.6)	
Birth weight			0.14
<750 g (*n* = 18, 17)	14 (77.8)	16 (94.1)	
750–1499 g (*n* = 94, 103)	26 (27.7)	22 (21.4)	
Antenatal corticosteroids received			0.24
No (*n* = 11, 3)	6 (54.6)	3 (100.0)	
Yes (*n* = 101, 116)	34 (33.7)	35 (30.2)	

*P* values are from tests of heterogeneity of the treatment effect on the relative risk scale, estimated using log binomial regression models fit using generalized estimating equations. The number (*N*) of infants within each subgroup are noted in parentheses in the first column. Incidence data for subgroup comparisons are shown as *N* (%).

### Secondary outcomes

The most common reason for treatment failure was respiratory distress requiring intubation (Table 3), with no between-group differences. Treatment failures were most likely to occur during the first hours after randomization (Supplemental Fig. 1). After day 2, fewer than half of the infants in both groups were undergoing ventilation. Among mother–infant dyads consented antenatally and started on CPAP at birth, we observed no differences between groups in rates of treatment failures (Seattle-PAP: 4/32, 12.5%; FP-CPAP: 10/35, 35.6%; *P* = 0.14). The duration of hospitalization was similar among infants in the Seattle-PAP (median 82 days, IQR 39–260) and FP-CPAP (median 82, IQR 37–285; *P* = 0.71) groups.

**Table 3:** Reasons for treatment failure and secondary outcomes.

Outcome	Seattle-PAP (*n* = 112)	FP-CPAP (*n* = 120)	Risk Difference (95% CI)	*P* value
Reason for treatment failure^a^				
Failure of successful transition^b^	15 (13.4)	18 (15.0)	−1.6 (−10.6–7.4)	0.73
Increase in fraction of inspired oxygen	15 (13.4)	22 (18.3)	−4.9 (−14.3–4.4)	0.30
Escalation of respiratory care requiring intubation^c^	22 (19.6)	24 (20.0)	−0.4 (−10.6–9.9)	0.95
Escalation of respiratory care to SiPAP	3 (2.7)	0 (0)	2.7 (−0.3–5.7)	0.11
Number of days on mechanical ventilation after trial entry, median (IQR)^d^	0 (0–4)	0 (0–5)	NA	0.20
Number of days any positive pressure support after trial entry, median (IQR)^e,f^	47.5 (34.5–75.5)	51 (32–74)	NA	0.99
Number of days of oxygen therapy after trial entry, median (IQR)^f^	28.5 (5–91)	34 (5–84)	NA	0.63
Number of days on room air after trial entry, median (IQR)^f^	46 (19–57)	43 (20–60)	NA	0.93
Number of days to achieve full enteral feeding after trial entry, median (IQR)^f,g^	12.5 (10–14)	11 (10–14)	NA	0.73
Number of days to achieve full-suck feeding, median (IQR)^f^	73 (63–80)	78 (66–83)	NA	0.95
Weight at discharge (kg)^f^	3.1 (2.6–3.8)	3.0 (2.6–4.1)	NA	0.89

Definition of room air includes infants who were on CPAP or nasal cannula with FiO2 of 0.21.

*SiPAP* synchronized inspiratory positive airway pressure, *IQR* Interquartile range, *NA* Not applicable.

^a^Treatment may have failed for more than one reason.

^b^Intubation within 72 h for surfactant administration after initiation of bn-CPAP and then fails to meet extubation criteria by 72 h.

^c^Intubation was recommended if more than two episodes of apnea requiring bag-mask ventilation were encountered or more than six episodes of apnea required stimulation in a 24 h period.

^d^Mechanical ventilation includes synchronized intermittent ventilation (SIMV) and high-frequency oscillatory ventilation (HFOV).

^e^Respiratory support included the use of nasal continuous positive airway pressure, SIMV, and HFOV.

^f^Infants who died before discharge were excluded (*n* = 8, Seattle PAP; *n* = 5, FP-CPAP).

^g^Full enteral and full-suck feeding defined as feeds >130 mL/kg/day.

### Adverse events, adherence to respiratory guidelines, and protocol violations

We observed no differences between the groups in the frequencies of pre-specified adverse events (Supplementary Table 1). No deaths or adverse events were attributable to either device. The causes of death included sepsis associated with disseminated intravascular coagulation (*n* = 5), necrotizing enterocolitis (NEC) refractory to medical/surgical care (*n* = 2), pulmonary hemorrhage (*n* = 2), respiratory failure (*n* = 2), withdrawal of support due to severe intraventricular hemorrhage (*n* = 1), and spontaneous intestinal perforation (*n* = 1). Adherence to respiratory guidelines was high; five cases of non-adherence centered around the use of nasal cannula, rather than CPAP, among infants requiring FiO_2_ > 0.21 prior to 32 weeks’ gestational age. Study protocol violations were typically administrative errors that resulted in the use of FP-CPAP in 7.1% (8/112) of infants assigned to Seattle-PAP and in the use of Seattle-PAP in 0.8% (1/120) of infants assigned to FP-CPAP. These violations were quickly corrected (<6 h) to limit exposure. Additional violations included transfer to a non-study site (*n* = 3) or physician request for FP-CPAP rather than assigned Seattle-PAP device (*n* = 1).

### Sensitivity analysis

We observed no differences in primary or secondary outcomes following the exclusion of infants (*n* = 5) with a delayed diagnosis of a major cardiac or lung malformation. In addition, we observed no differences in primary or secondary outcomes following the exclusion of infants (*n* = 13) with protocol deviations.

## Discussion

Preclinical studies showed that Seattle-PAP provides more variable pressure oscillations, along with a broader range of frequencies and with a shift to lower frequencies of pressure oscillations, than traditional bubble CPAP [11, 23]. These properties suggested that Seattle PAP would improve air exchange and better address the stochastic properties of newborn infants’ lungs [8]. Initial clinical studies among larger, more mature infants with lower supplemental oxygen requirements than infants in the present study, reported that the effort of breathing was lower in the infants supported with Seattle-PAP than with traditional bubble CPAP [12]. The lack of beneficial effects of Seattle PAP in the population in the present studies suggests a number of possible interpretations.

In premature infants, respiratory failure is a complex disorder, variously attributed to structurally and functionally immature lungs, compliant chest walls, obstructive and/or central apnea, alone or in combination [8, 24, 25]. While the primary outcome (CPAP failure) is consistent with previous studies [18, 26], we acknowledge the need for intubation and mechanical ventilation (CPAP failure) may not reflect primary respiratory failure. To that end, CPAP failure can reflect a variety of etiologies, including airway obstruction, nasal obstruction, and gastric distention [20]. In contrast to adult respiratory failure, identifiable phenotypes for respiratory failure, based on clinical criteria or biomarkers, are not readily available in preterm infants [27]. While no evidence of heterogeneity of treatment effect across the reasons for treatment failure were observed, the study was not powered to detect potential differences among subgroups. Further studies may identify subgroups of infants who might have greater responses to Seattle-PAP than were observed in our study, such as the earlier studies on Seattle-PAP that involved larger, more mature infants with respiratory failure largely attributable to primary respiratory dysfunction [8, 12]. In following the common practice of alternating the interface used to deliver CPAP between nasal prongs and mask, designed to minimize nasal injury, inconsistent delivery of distending pressures may have dampened the Seattle-PAP pressure fluctuations and obscured any differences between the two groups [9, 12].

The strengths of this study include participation by a network of five NICU sites that share a consistent approach to the care of premature infants [14]. Rates of parental/caregiver consent were high (>80%) and reflect a motivated group of health care providers and families. The variety of included sites (size, location, and academic/private) increases the generalizability of findings. Absence of any observable differences in rates of pneumothorax, NEC, or other adverse effects, associated with use of Seattle-PAP is notable. Without clear advantages of either therapy, health care providers may choose the device that is associated with less resource utilization or greater convenience [28]. To inform decisions about resource allocation, a formal economic evaluation is ongoing [29].

In part, Seattle-PAP was designed and developed to address high rates of CPAP failure among low-and middle-income countries (LMIC) [8, 12]. The design and context of the present study has limited applicability to LMIC settings; thus, observed findings should not preclude the execution and conduct of a large, RCT of Seattle-PAP in LMIC. In fact, consistent with the calls from previous investigators, such trials are critically necessary to better characterize the skills, organization, and resources necessary to optimize bubble CPAP in LMIC [30–32].

Our study has several limitations. The current study used a superiority design; thus, findings cannot be interpreted to show noninferiority of Seattle-PAP to FP-CPAP. Since the study was conducted at a single academic center, the primary outcome was short-term treatment efficacy (CPAP treatment failure), rather than death or morbidity (BPD). Although our interventions did not permit blinding of treatment assignment to health care providers, thresholds for respiratory failure were based on pre-specified, objective criteria to minimize bias [13]. Although care might have been affected by knowledge of treatment allocation, outcome assessors were not privy to group allocation. Meticulous attention to optimize device positioning in the current study likely contributed to low rates of device-related complications (e.g., nasal injury), but the requisite skills to optimize bubble CPAP may not be routine in many health care systems.

Regardless of treatment assignment, CPAP failure rates were high among infants born at <27 weeks, particularly those born at ≤24 weeks of gestation, and additional therapeutic strategies are needed [9]. Previous well-designed, large RCTs evaluating CPAP as a primary mode of respiratory support reported rates of CPAP failure of 46 and 51%, respectively [5, 6]. While rates of CPAP failure in the present study are lower than those of previous trials, differences in clinical characteristics (gestational age, use of surfactant) and definitions for CPAP failure (timing, thresholds) preclude meaningful comparisons [5, 6]. Markedly variable CPAP failure rates among centers reflect a number of determinants, including time and experience with CPAP, and the level and extent of multidisciplinary collaboration [9, 33]. Previous investigators have characterized pragamatic, interdisciplinary strategies to reduce CPAP failure rates, with the ultimate goal of reducing rates of chronic lung disease and improving neonatal outcomes [20].

Even though more than 230 preterm infants were enrolled, rates of many primary outcomes were low, limiting our statistical power to detect differences between groups. Although patients were only eligible to enter the study if they were admitted to one of the participating NICUs within 72 h of birth, adjustment could not be made for care provided prior to transfer, which is a possible confounder in the analysis.

In conclusion, among premature infants born at <30 weeks of gestation, we observed no differences between Seattle-PAP and FP-CPAP in rates of CPAP treatment failure, mortality, morbidity, or the duration of hospitalization. The absence of any adverse events associated with Seattle-PAP is notable, wherein potential differences in resource utilization between the two devices must be considered. Additional studies are needed to determine whether Seattle-PAP could prove to be useful in offering practical respiratory support in resource-limited health care settings.

## Supplementary information

Supplementa Fig 1

Supplementary Table 1: Adverse events

supplementary figure legend

## Data Availability

The data will be made available from the global data-sharing enterprise Vivli (https://vivli.org/resources). The Vivli platform provides an independent data repository, an in-depth search engine, and a secure cloud-based analytics platform.
