# Supplementary figures and images for: A trial comparing continuous positive airway pressure (CPAP) devices in preterm infants
Source: J Perinatol. 2020 May 20;40(8):1193–201. doi: 10.1038/s41372-020-0690-5 (PMC7375950; doi:10.1038/s41372-020-0690-5)

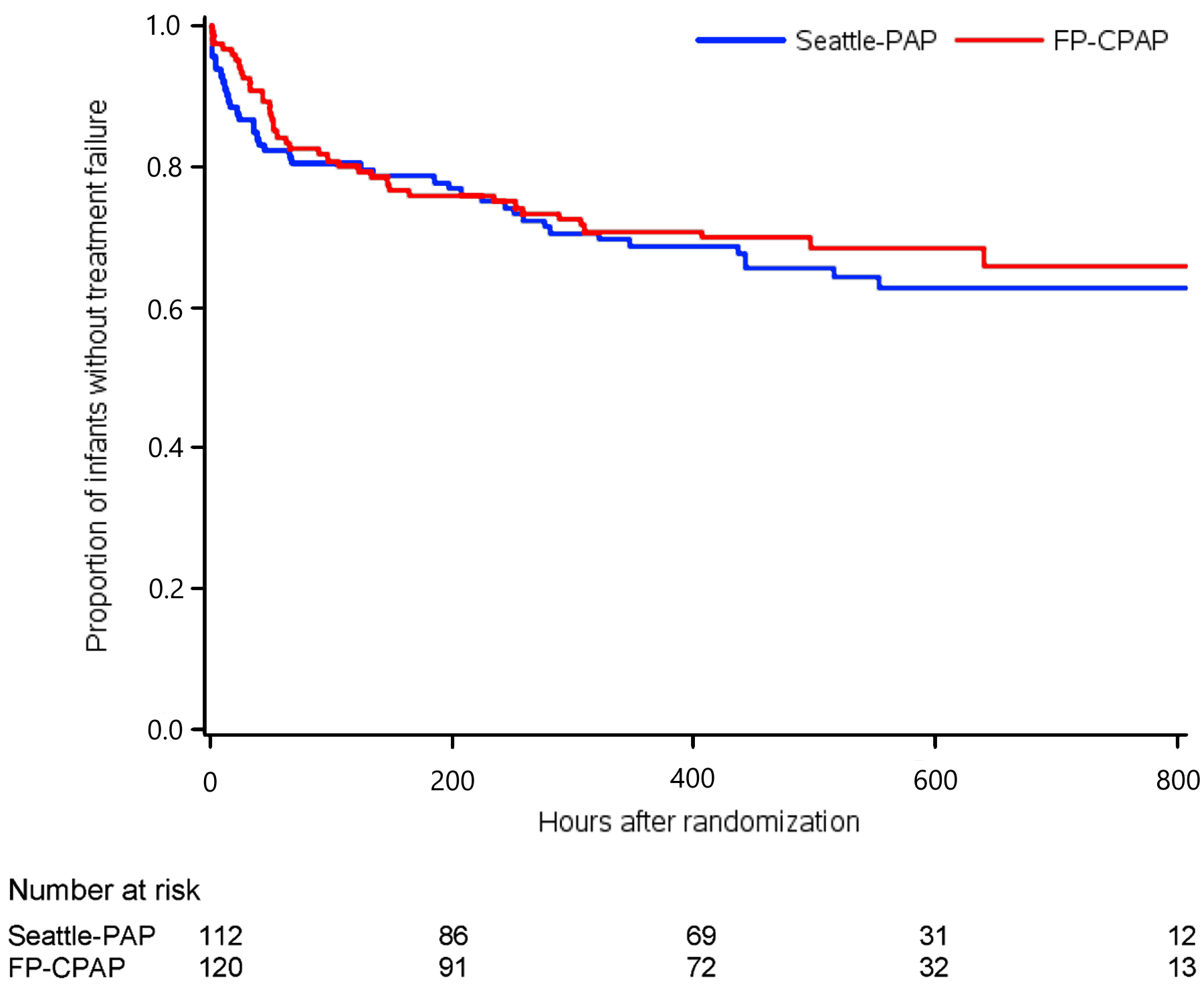

Supplement: Supplementary file 1 — Supplementa Fig 1 [file 41372_2020_690_MOESM1_ESM.jpg]
