# Supplementary material for: A trial comparing continuous positive airway pressure (CPAP) devices in preterm infants
Source: J Perinatol. 2020 May 20;40(8):1193–201. doi: 10.1038/s41372-020-0690-5 (PMC7375950; doi:10.1038/s41372-020-0690-5)
Supplement: Supplementary file 2 — Supplementary Table 1: Adverse events [file 41372_2020_690_MOESM2_ESM.pdf]

Supplementary Table 1: Adverse Events

	**Seattle-PAP (N=112)**	**FP-CPAP (N=120)**	**Risk Difference (95% CI)**	**P-value**
Death before discharge[1]	8 (7.1)	5 (4.2)	3.0 (-3.0 to 8.9)	0.32
Oxygen supplementation at gestational age of 36 weeks[1,2]	35 (33.7)	46 (40.0)	-6.4 (-19.1 to 6.4)	0.33
Discharged home with oxygen[2]	33 (31.7)	43 (37.4)	-5.7 (-18.2 to 6.9)	0.38
Glucocorticoid treatment for lung disease after trial entry[3]	32 (28.6)	36 (30.0)	-1.4 (-13.1 to 10.3)	0.81
Pneumothorax after trial entry	4 (3.6)	5 (4.2)	-0.6 (-5.6 to 4.4)	1.00
Pulmonary interstitial emphysema after trial entry	4 (3.6)	5 (4.2)	-0.6 (-5.6 to 4.4)	1.00
Nasal trauma after trial entry	7 (6.3)	6 (5.0)	1.3 (-4.7 to 7.2)	0.68
Confirmed sepsis after trial entry[4]	11 (9.8)	13 (10.8)	-1.0 (-8.8 to 6.8)	0.80
Definitive patent ductus arteriosus closure	2 (1.8)	1 (0.8)	1.0 (-2.0 to 3.9)	0.61
Necrotizing enterocolitis grade 2 or 3[5]	13 (11.6)	11 (9.2)	2.4 (-5.4 to 10.3)	0.54
Isolated intestinal perforation after trial entry	0 (0)	2 (1.7)	-1.7 (-4.1 to 0.7)	0.50
Laser surgery for retinopathy of prematurity[6]	3 (2.9)	4 (3.5)	-0.6 (-5.2 to 4.1)	1.00
Cystic periventricular leukomalacia	17 (15.2)	22 (18.3)	-3.2 (-12.8 to 6.4)	0.52
Intraventricular hemorrhage grade 3 or 4	13 (11.6)	17 (14.2)	-2.6 (-11.2 to 6.1)	0.56

Positive values favor the Seattle-PAP group, negative values favor the high-flow group; CI: confidence interval.

[1] All in-hospital deaths occurred prior to 36 weeks.

[2] Infants who died before discharge were excluded (N=8, Seattle PAP; N=5, FP-CPAP).

[3] Includes infants treated with systemic glucocorticoids (e.g., dexamethasone) for primary lung disease.

[4] The criteria for confirmation of sepsis were a positive blood culture and treatment with intravenous antibiotics for 48 hours or longer.

[5] Modified Bell's criteria stage ranges from I to III, with higher stages indicating greater disease severity.

[6] Includes use of ablative laser photocoagulation and/or intravitreal injection of vascular endothelial growth factor (VEGF) inhibitor (e.g., bevacizumab).

Note: All definitive PDA closures were via percutaneous techniques.
