# Supplementary material for: A trial comparing continuous positive airway pressure (CPAP) devices in preterm infants
Source: J Perinatol. 2020 May 20;40(8):1193–201. doi: 10.1038/s41372-020-0690-5 (PMC7375950; doi:10.1038/s41372-020-0690-5)
Supplement: Supplementary file 3 — supplementary figure legend [file 41372_2020_690_MOESM3_ESM.docx]

Supplementary Figure Legend

Figure 1, Supplemental: Infants without Treatment Failure after Randomization

Shown are the percentages of infants (<30 weeks of gestation, <1500 grams), in whom treatment with Seattle-PAP or Fisher-Paykel CPAP (FP-CPAP) did not fail after extubation. Rates of CPAP treatment failure occurred in 40 of 112 infants (35.7%) in the Seattle-PAP group and 38 of 120 (31.7%) in the FP-CPAP group (risk difference, 4.1 percentage points; 95% CI, -8.1 to 16.2; P=0.51). CPAP denotes continuous positive airway pressure.
